# Near-Infrared Spectroscopy Combined with Explainable Machine Learning for Storage Time Prediction of Frozen Antarctic Krill

**DOI:** 10.3390/foods14081293

**Published:** 2025-04-08

**Authors:** Lin Li, Rong Cao, Ling Zhao, Nan Liu, Huihui Sun, Zhaohui Zhang, Yong Sun

**Affiliations:** 1 Yellow Sea Fisheries Research Institute, Chinese Academy of Fishery Sciences, Qingdao 266071, China; lilin_4x@163.com (L.L.); caorong@ysfri.ac.cn (R.C.); zhaoling@ysfri.ac.cn (L.Z.); liunan@ysfri.ac.cn (N.L.); sunhh@ysfri.ac.cn (H.S.); 2 College of Food Science and Engineering, Ocean University of China, Qingdao 266100, China

**Keywords:** near-infrared spectroscopy, interpretable machine learning, light gradient boosting machine, Antarctic krill, storage time

## Abstract

Antarctic krill (*Euphausia superba*) represents a promising sustainable protein source for human consumption. While a portion of the catch undergoes immediate onboard processing, the majority is preserved as frozen raw material, with storage duration significantly impacting product quality and safety. This study established a novel approach for rapid quality assessment through storage time prediction. Traditional chemical quality indicators of krill during a 12-month storage were first monitored and the correlation between the quality and storage time was verified. Coupled with four different regression machine learning algorithms, near-infrared spectroscopy (NIRS) was applied to develop models. Following optimal spectral preprocessing selection and hyperparameters optimization, the light gradient boosting machine (LightGBM) model yielded the best storage time prediction performance, with the R^2^ of the test set being 0.9882 and the errors RMSE, MAE, and MAPE being 0.3724, 0.2018, and 0.0431, respectively. Subsequent model interpretation results revealed a strong correspondence between model-related NIR features and chemical indicators associated with quality changes during krill frozen storage, which further justified the model’s predictive capability. The results proved that NIR spectroscopy combined with LightGBM could be used as a rapid and effective technique for the quality evaluation of frozen Antarctic krill, offering substantial potential for industrial implementation.

## 1. Introduction

Antarctic krill (*Euphausia superba*, hereafter referred to as krill) represents one of Earth’s most abundant biological resources, with an estimated biomass of 300–500 million tons [1]. Krill is a rich source of high-quality protein, with the advantage over other animal proteins of being low in fat and a rich source of omega-3 fatty acids [2,3]. Therefore, as a sustainable and renewable food source, krill has considerable potential to meet human nutritional needs in the future with good public acceptance [4]. In modern krill fisheries, in addition to some krill that are processed immediately onboard the catching vessel into krill meal and krill oil, a sizable amount of the krill is frozen and then transshipped to land for further processing [5,6,7]. However, protein denaturation and oxidized lipid degradation can be easily caused by autolytic enzymes, temperature fluctuations, storage time, and other factors throughout the low-temperature storage and transit process, leading to a decrease in krill quality [8,9]. To maintain the safety and quality of krill, the krill industries must monitor and predict quality changes during storage. It is critical to identify the key signs and develop a shelf-life prediction model for frozen krill.

The quality of krill is typically characterized using conventional physical and chemical indicators, including total volatile base nitrogen (TVB-N) [9], non-protein nitrogen (NPN) [10], phospholipids (PLs) [11] and free fatty acid (FFA) [12], etc. NPN, encompassing nitrogenous compounds excluding macromolecular proteins, serves as a sensitive indicator of protein degradation. TVB-N, a subset of NPN, mainly refers to volatile organic amines generated by the further decomposition of proteins and amino acids under the action of enzymes during krill frozen storage [13]. The unique presence of eicosapentaenoic acid (EPA) and docosahexaenoic acid (DHA) as PLs in krill oil [14] makes these compounds particularly susceptible to oxidative degradation, while enzymatic activity promotes lipid breakdown into FFA. Therefore, NPN and TVB-N were employed as indicators of protein and amine degradation, whereas PL and FFA were employed as biomarkers of lipid degradation. Despite their reliability and sensitivity, the shortcomings of these conventional methods are also prominent, such as labor-intensive pretreatment steps, lengthy operations, high costs, and the requirement for skilled operators [15,16]. Thus, it would be desirable to employ an accurate, rapid, and non-contaminating measurement technique for the quality control of krill and its processed products.

In recent years, near-infrared (NIR) spectroscopy has gained wide acceptance within the food industry for raw material testing, product quality control, and process monitoring due to its effectiveness and efficiency. NIR spectroscopy deals with the interaction of matter and light in the near infrared region of the electromagnetic spectrum between 12,500 and 4000 cm^−1^ (800–2500 nm) [17], containing rich information about the composition and molecular structure of most types of organic compounds. In addition, the physical properties or characteristics of the sample can also be reflected by the NIR spectrum [18,19], making the technique even more suitable for the analysis of food quality. The main advantages of NIR spectroscopy over other analytical techniques also include the ease of sample preparation with little or no pre-treatment, fast determination speed, low cost, simplicity of operation, and so on [20].

The integration of machine learning (ML) with NIR spectroscopy has revolutionized analytical capabilities in food science [21]. The primary objective of ML is to make it possible for computer systems to build analytical models on their own without the need for explicit programming, with strong generalization, accurate prediction, and quick processing times [22]. Several ML algorithms, such as partial least squares regression (PLSR), support vector regression (SVR), and random forest (RF), have been widely used. Currently, the fields of studies have combined NIR and ML to figure out aquatic product quality [23,24,25], identify tampering [26], trace its origin [27], and classify its species [28,29]. To determine the storage duration, the technique is primarily utilized for grains [30,31], fruits [32,33,34], nuts [35], and vegetables [36], with less application in the case of aquatic items.

Generally speaking, most complex machine learning models are of the black-box type, making it difficult to account for the model’s mechanism of decision-making in a human-understandable way [37]. In order to overcome the drawback, a collection of procedures and techniques known as explainable artificial intelligence (XAI) have been developed to aid in describing model correctness, fairness, transparency, and results [38,39]. For the models based on NIR spectroscopy, applications for XAI have already been found in the quality evaluation of food [40,41,42,43].

The present study aimed to develop a novel approach for predicting the storage time of frozen krill through the integration of NIR spectroscopy and ML. Specifically, the key quality indicators of krill during a 12-month storage were monitored, and the correlations of those indicators with storage time were analyzed. NIR and a variety of machine learning algorithms were utilized to build regression models. By spectra preprocessing, model training and testing, as well as hyperparameter-tuning, an optimal model was established for the rapid identification of krill storage time, which could indirectly assess the quality of the krill. Model interpretation was subsequently performed to explore the relevant NIR signals that closely connected with model regression and, thus, justify the appropriateness and reliability of the model.

## 2. Materials and Methods

**Figure 1:**
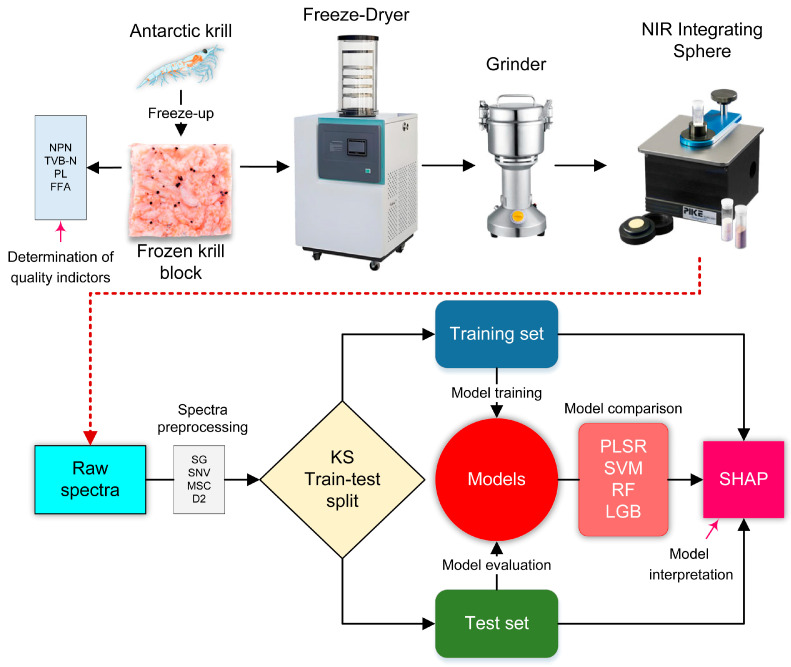
Main framework of the proposed methodology.

The flowchart shown in Figure 1 exhibits the main framework of the proposed methodology of this study. Detailed information about methods and materials are described as follows.

### 2.1. Materials

Krill used in this work were captured in the Antarctic Sea Area 48.1 by China National Fisheries Corp (Beijing, China). They were quick-frozen as krill blocks onboard. Upon arrival at the laboratory, the frozen krill were cut into smaller blocks of 20 cm × 15 cm × 8 cm size, with a mass of between 500 and 600 g. The blocks were then placed in a sample bag and kept at −20 °C for storage. During a period of 12 months, two randomly selected blocks were sampled monthly for quality indicator determination and NIR spectra acquisition.

### 2.2. Measurement of Quality Indicators

Four krill quality indicators were measured each month in this study: TVB-N, NPN, FFA, and PL. According to Kolakowski [10], the NPN content is the trichloroacetic acid (TCA) soluble nitrogen of krill homogenate, which was determined by the Kjeldahl method, and the results were represented as mg/g. The TVB-N content was determined by the trace diffusion method, which can refer to Chinese standard GB 5009.228-2016 [44], and the results were represented as mg/100 g. To determine the content of PL and FFA, the lipid in krill was first extracted by rotary evaporation using the chloroform–methanol method. The content of PL was determined with the molybdenum blue colorimetric method (Chinese national standard GB/T 5537-2008 [45]), and FFA content was determined with the method of Lowry et al. [46]. Each indicator was measured in triplicate, and all results were expressed as mean ± standard deviation (SD).

In addition, Pearson correlation analysis was conducted using Graphpad Prism (version 9.5.0) to illustrate the relationship between the content of each quality indicator and storage time (statistically significant correlation at *p* < 0.05).

### 2.3. NIR Spectra Acquisition

To enhance the signal intensity and reduce moisture interference, the krill samples were first lyophilized by a lyophilizer (Beta 1-8 LD plus; Christ, Osterode, Germany) at −60 °C, 0.098 MPa, and ground into powder. About 2–3 g of formed krill powder was placed in a glass sample vial, and fifty such vials were prepared each month for the NIR spectra acquisition.

The measurement of NIR spectra was performed using a Tensor 27 spectrometer (Bruker Optic, Karlsruhe, Germany) equipped with a broadband beam-splitter and a NIR-integrating sphere (IntegratIR^TM^; PIKE, Charlotte, NC, USA). The spectrometer was warmed up for 30 min before spectra gathering. Each spectrum was the average of 64 scans of the same sample. The spectral data were recorded across the wavenumber range 10,000–4000 cm^−1^ with a resolution of 4 cm^−1^. The ambient conditions were maintained at a constant temperature (25 °C) and relative humidity throughout the measurement. System calibration was performed using a diffuse gold plate reference, with instrumental control and data acquisition managed through OPUS (version 7.0) software.

### 2.4. Spectra Preprocessing

The recorded NIR spectrum may be impacted by undesired scattering effects (baseline shift and nonlinearity) because the wavelength of NIR electromagnetic radiation is similar to the particle size in biological samples. For this reason, proper preprocessing is crucial for further modeling. In this study, spectral preprocessing approaches including multivariate scattering correction (MSC); standard normal variable (SNV) transformation; Savitzky–Golay smoothing (SG); second-order derivative (D2); and the combination of D2 with MSC, SNV, and SG.

### 2.5. Machine Learning Models

The effective implementation of NIR spectroscopy as a robust analytical tool fundamentally depends on advanced computational and data analysis techniques. In this study, several machine learning algorithms in conjunction with NIR spectroscopy were implemented to develop robust predictive models. The specific algorithms employed are comprehensively described in the following sections.

#### 2.5.1. Partial Least Squares Regression (PLSR)

PLSR is a multivariate data analysis method in statistics and machine learning, particularly suitable for handling situations where there is multicollinearity between dependent and independent variables [47]. Unlike principal component regression (PCR), PLSR simultaneously considers both predictor and response variables during dimensionality reduction, with a view to minimizing the dimensionality while maximizing the prediction performance, making it perform exceptionally well in high-dimensional data and small-sample problems [32].

#### 2.5.2. Support Vector Regression (SVR)

SVR is an extension of support vector machines (SVMs), which have been shown to be a useful tool for estimating real-value functions [48]. SVR is a supervised-learning technique that uses a symmetrical loss function for training, penalizing both overestimations and underestimations, ensuring balanced error treatment. The core mechanism of SVR involves the creation of an ε-insensitive tube around the estimated function, where predictions falling within a predetermined threshold (ε) are considered acceptable and incur no penalty. This ε-insensitive technique effectively filters out minor deviations while focusing on significant prediction errors outside the tolerance boundary. The method’s computational efficiency and strong generalization capabilities make it particularly suitable for high-dimensional spectral data.

#### 2.5.3. Random Forest (RF)

RF is an ensemble machine learning algorithm using bagging and decision tree concepts [49], excelling in handling high-dimensional data. The algorithm operates through randomly selecting samples from the training set of data in a relaxed manner, and the resampling technique is used to create a new training dataset. Next, the decision trees are divided into nodes to obtain the corresponding output values. Finally, the output values of all the decision trees are averaged (for regression) or voted upon (for classification), with the resulting average representing the predicted value of the RF regression model and the resulting voting score representing the sample’s classification outcome [50].

#### 2.5.4. Light Gradient Boosting Machine (LightGBM)

LightGBM, developed by Ke et al. at Microsoft [51], is an effective gradient boosting framework that makes use of tree-based learning techniques. To improve prediction accuracy, it builds several decision trees in a sequential manner. The model stands out for its efficient data structures, multi-threading, minimal memory footprint, and histogram-based algorithms, among other optimized techniques. LightGBM can handle complex models and process huge datasets quickly thanks to these features. The algorithm is a favored approach for many machine learning tasks due to its efficiency and adaptability.

### 2.6. Model Construction and Evaluation

The dataset was partitioned into training and test sets (2:1 ratio) using the Kennard–Stone algorithm, ensuring representative sampling across the spectral space [52]. Model performance was evaluated using four metrics: coefficient of determination (R^2^), root mean square error (RMSE), mean absolute error (MAE), and mean absolute percentage error (MAPE).

### 2.7. Hyperparameters Optimization

Optuna was used in this work to optimize each model’s hyperparameters further to improve the performance of the machine learning models, as each model’s performance is strongly correlated with its hyperparameter settings. In 2019, Akiba et al. [53] published the Optuna algorithm, a revolutionary algorithm that performs hyperparametric optimization automatically and introduces new design criteria in the field of hyperparametric optimization. Optuna leverages cutting-edge algorithms for sampling hyperparameters and pruning unpromising trials effectively, enabling rapid identification of optimal parameter configurations [54].

The Optuna algorithm performs with greater advantages over previous hyperparametric algorithms (e.g., genetic algorithm, grid search, stochastic search). These advantages include being easy to save, remembering the entire optimization process, strong visualization, etc., which are useful for quick selection of the optimal hyperparameter combination.

### 2.8. Model Interpretation

As a model interpretation technique based on game theory, SHAP (SHapley Additive exPlanations, version 0.46.0) analysis aids in our comprehension of the rationale behind the model predictions. According to Lundberg et al. [38], SHAP is a local diagnostic that determines the contribution of each feature’s values to the model’s anticipated outcomes, generating an explanatory score for each feature. Based on the idea of Shapley values, which are ranked and added together to determine the impact of each feature on the model’s output, SHAP values are calculated. This method may interpret feature significance scores from intricate training models as a single framework for interpreting predictions and provides an interpretable prediction for a test sample.

### 2.9. Computing Implementation

Machine learning model building and hyperparameter optimization were implemented with Python (version 3.11) and relative scientific packages. The codes for spectral preprocessing, PLSR, SVR, RF, LightGBM, Optuna, and SHAP algorithms were all obtained from GitHub.

## 3. Results and Discussion

### 3.1. Correlation Between Krill Quality Indictors and Storage Time

**Figure 2:**
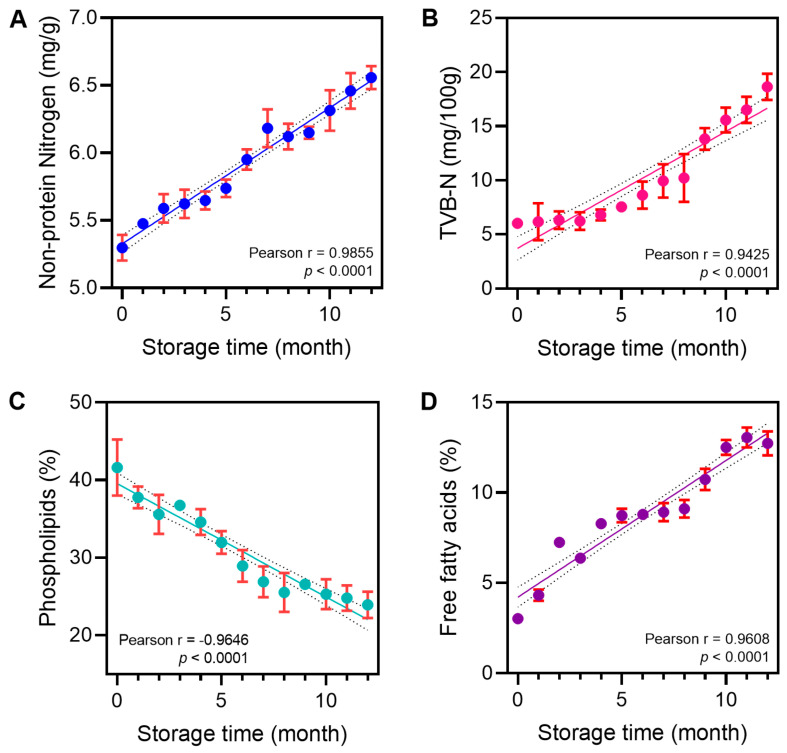
Changes in quality indicators ((**A**) NPN; (**B**) TVB-N; (**C**) PL; (**D**) FFA) and correlation analysis during the frozen storage of krill.

It has long been proved that krill muscle proteins are denatured easily during frozen storage due to endogenous proteolysis [55]. As shown in Figure 2A,B, both NPN and TVB-N showed a continuous increasing trend during the 12-month frozen storage period, which is consistent with the results of Huang et al. [56], simultaneously demonstrating strong positive correlations with storage time (r = 0.9855 and 0.9425, respectively; *p* < 0.0001). During frozen storage, phospholipases hydrolyze PLs to generate free fatty acids, lysophospholipids, and diacylglycerol [57]. From the changes of PLs in Figure 2C, it can be seen that PL showed a stable decreasing trend throughout the storage period, and the Pearson correlation analysis showed r = −0.9646 (*p* < 0.0001), indicating that phospholipids were continuously decomposed during the storage process, which coincided with the trend of the increase in the content of FFA (Figure 2D; r = 0.9608, *p* < 0.0001). In Bao’s study, the FAA content of frozen krill also steadily increased throughout the six-month frozen storage period [58]. It can be observed from the findings that there was a significant correlation between the four quality indicators and the storage time. Therefore, it is feasible to evaluate krill quality by determining or predicting frozen storage time.

### 3.2. Preliminary Analysis of Krill Spectra

**Figure 3:**
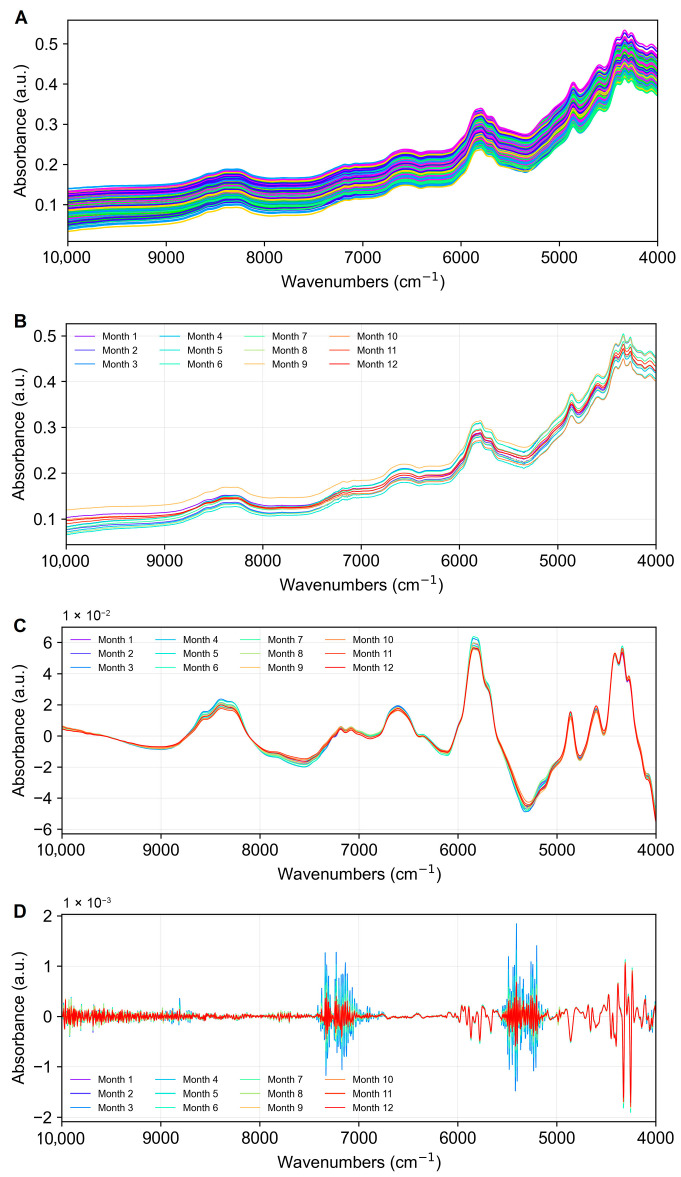
NIR spectra of the krill sample set with different frozen storage time. (**A**) Raw NIR spectra of all samples. (**B**) Monthly average spectra. (**C**) Baseline-corrected average spectra. (**D**) Second-derivative transformed average spectra.

In this study, a total of 600 NIR spectra were collected, i.e., 50 NIR spectra per storage month. The raw NIR spectra of frozen–dried krill powder are displayed in Figure 3A. As can be seen from the figure, all of the samples have the same general profile regarding their spectral trends, despite some absorbance intensity variations among the samples. It is noted that the sample’s absorbance clearly exhibits absorption peaks in the wave number ranges of around 8400 cm^−1^, 5800 cm^−1^, and 5000 cm^−1^ to 4000 cm^−1^, which are caused by the frequencies of the stretching vibrations of C-H, N-H, and C=O bonds tripling and merging [59]. Furthermore, the absorbance and general trend of the spectra varied as a result of different preprocessing methods (Figure S1), which is related to the noise reduction and baseline correction effect of preprocessing on the spectra.

Figure 3B shows the average spectra of krill samples for each month during the 12-month storage period. In order to eliminate baseline drift, which causes changes in the position or intensity of the peaks and to show the variability of the spectra from month to month, a baseline correction was applied to the 12-month average spectra by polynomial fitting, and the results are shown in Figure 3C. As can be seen in Figure 3C, the average spectra of krill samples from different months differed in several bands. In order to further enhance the subtle variations of the spectra, the second-order derivatives of the average spectra were processed, and the results are shown in Figure 3D, which clearly shows that the average spectra of different months have obvious absorption differences in the wave number ranges of 7400–6800 cm^−1^ and 5600–5100 cm^−1^.

### 3.3. Prediction Modeling

#### 3.3.1. Optimal Spectral Preprocessing Selection

Based on raw and different preprocessed spectra, four different machine learning algorithms (PLSR, SVR, RF, and LightGBM, with default hyperparameters listed in Table S1) were employed to map the NIR spectral data. The modeling results are shown in Table 1. From the evaluation results in the table, it is evident that all machine learning regression models constructed using preprocessed spectra perform better than those using the raw ones. This outcome demonstrates that the spectral feature mapping generated by the algorithm is significantly impacted by spectral noise and measurement artifacts arising from instrumental variations and environmental interference factors. The model’s performance can be substantially enhanced with appropriate preprocessing.

As Table 1 shows, the most efficient preprocessing method for SVR models is D2 plus SNV (D2+SNV), while D2+SG is the best preprocessing method for PLSR, RF, and LightGBM models. These two spectral preprocessing approaches consistently resulted in the maximum R^2^ for all models while maintaining minimal error values. Studies have proved that appropriate integration of multiple preprocessing techniques can significantly improve model predictive performance [60,61,62]. Notably, the LightGBM model performed the best out of all the models created with proper spectral preprocessing, yielding the highest test set R^2^ of 0.9864 and relatively lower error values (RMSE = 0.3724, MAE = 0.2018, MAPE = 0.0431). It is also evident to conclude that ensemble learning techniques are better suited for handling high-dimensional data when employing the entire spectrum compared to the conventional chemometric approaches such as PLSR and SVR, as also mentioned by Mienye et al. [63].

#### 3.3.2. Model Hyperparameters Optimization

**Figure 4:**
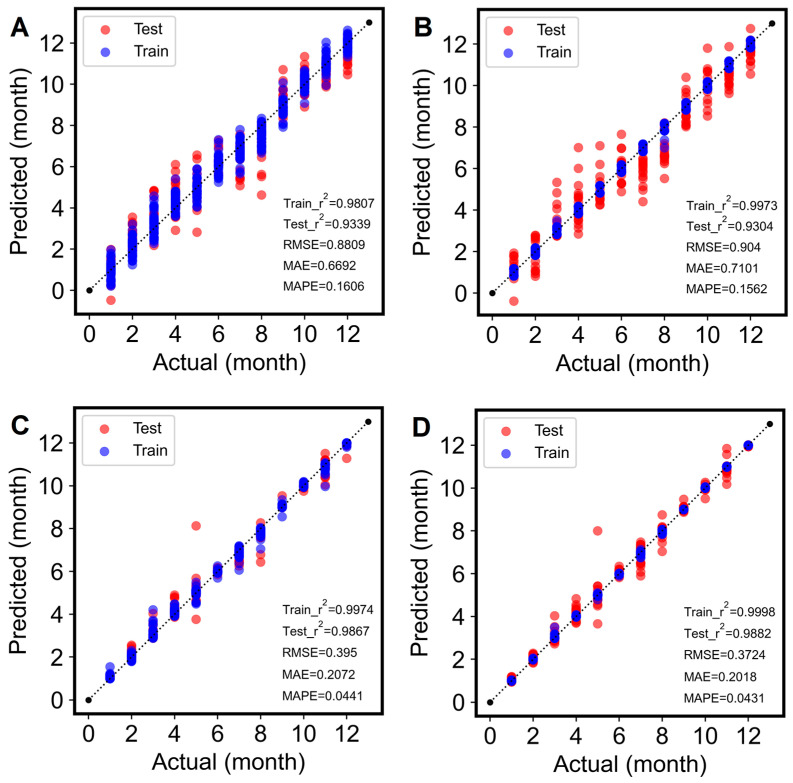
Optuna-optimized regression modeling of storage time of krill ((**A**) PLSR; (**B**) SVR; (**C**) RF; (**D**) LightGBM).

To further improve the model’s performance, the Optuna algorithm was applied to optimize the important hyperparameters of all four models with corresponding preprocessing. The specific optimization procedures are illustrated in Figure S2, and the obtained optimal hyperparameter combinations for each model are listed in Table S1. The performance of the tuned prediction models, including the R^2^ of the training set and test set as well as the errors, is shown in Figure 4. As exhibited in Figure 4, the performance of the models tuned by Optuna is enhanced to varied degrees in comparison to those using the default hyperparameters, which is consistent with the findings reported by Zhou et al., who utilized the Optuna algorithm to optimize XGBoost hyperparameters for developing a predictive model [25]. Specifically, for traditional chemometrics or machine learning methods, hyperparameter tuning can significantly improve model performance. The test set R^2^ value of the PLSR model was improved from 0.8211 to 0.9339 after Optuna tuning, and for the SVR, the R^2^ was improved from 0.8240 to 0.9304; the corresponding errors were also lowered. However, for ensemble learning models, good model performance can already be achieved with default hyperparameters, so hyperparameter optimization has a relatively limited impact on improving model performance. The R^2^ of the RF and LightGBM were slightly improved to 0.9867 and 0.9882, respectively.

Overall, the R^2^ values of all four proposed models with optimized hyperparameters exceeded 0.9, which on the one hand indicates the good performance of the models used and, on the other hand, shows that there is an obvious regression trend in the spectral data itself. Based on the analysis and comparison, it can also be seen that the PLSR model has the greatest performance increase among the models after hyperparameter tuning. Above all, the LightGBM achieved the best performance with the highest R^2^ value, both with training (0.9998) and test sets (0.9882). The RMSE, MAE, and MAPE are 0.3724, 0.2018, and 0.0431, respectively. This is probably due to the fact that LightGBM applies more sophisticated techniques compared with RF, such as gradient-based one-side sampling, exclusive feature bundling, and histograms, to achieve more efficient and accurate modeling of high-dimensional data [51].

### 3.4. Interpretation of the Optimal Model

**Figure 5:**
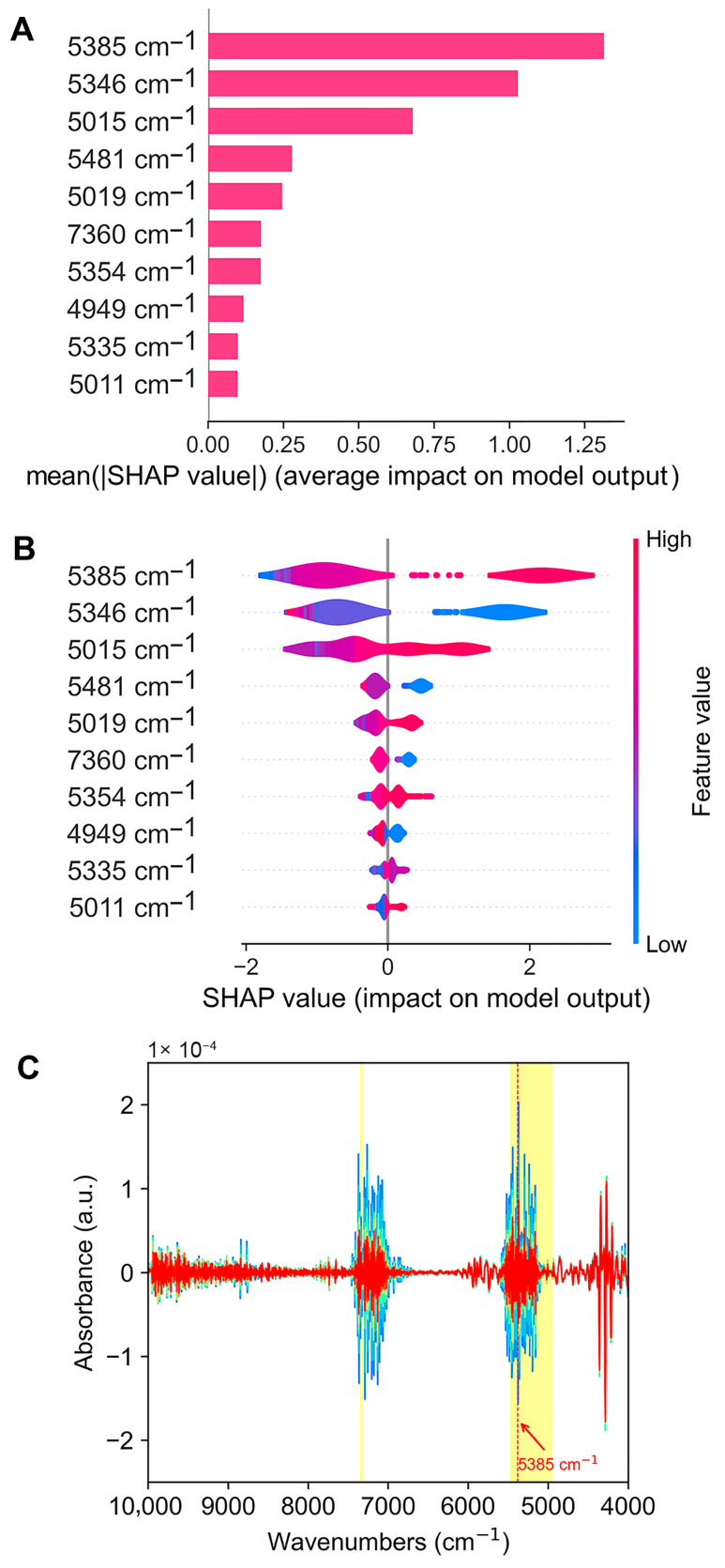
SHAP interpretation results of LightGBM model. (**A**) Bar chart of the mean absolute SHAP values of top 10 features. (**B**) Violin plot of SHAP values of top 10 features. (**C**) Second-order derivative infrared bands corresponding to the top 10 features.

To obtain an overview of which features are the most important for the LightGBM model, and to further verify the rationality of the black-box model in predicting krill storage time, we used the SHAP algorithm in conjunction with the dataset to interpret the model. The interpretation results are shown in Figure 5.

In the bar chart of Figure 5A, we arranged the average absolute SHAP values of the top 10 features in descending order. The larger the absolute value, the more important the feature is for the model’s prediction. It can be seen that the near-infrared features closely related to the prediction of krill storage time by LightGBM are mainly concentrated in the range of 5000–5500 cm^−1^ and also include 7360 cm^−1^; among these, the near-infrared absorption at 5385 cm^−1^ contributes the most, followed by 5346 cm^−1^ and 5015 cm^−1^. The violin plot of Figure 5B gives further information about the influence direction of features on the model output. Figure 5B shows that among the top 10 features, 5385 cm^−1^, 5015 cm^−1^, 5019 cm^−1^, 5354 cm^−1^, 5011 cm^−1^, and 5335 cm^−1^ have a positive impact on the model’s output, meaning that the higher the infrared absorption of these features, the longer the predicted storage time by the model. Conversely, the higher the absorption at 5346 cm^−1^, 7360 cm^−1^, 5481 cm^−1^, and 4949 cm^−1^, the shorter the predicted storage time by the model.

Because the overtone and combination absorption bands of chemical bond vibrations frequently overlap, the spectra in the near-infrared range are typically rather complicated, making it challenging to identify the absorbing groups qualitatively. Plus, the complexity of the krill’s biological matrix makes analysis even more difficult. Therefore, only a general analysis can be performed based on the model’s explanatory information. As the literature about NIR band assignments has noted [64,65,66], in the NIR region of 5000–5500 cm^−1^ (as shown in Figure 5C), there is the combination of NH stretching and NH bending of primary amines(-NH_2_) as well as the combination of NH stretching of primary amides (-CONH_2_). In addition, the combination of NH stretching, amide, and the second overtone of the amide of secondary amides (-CONH-), the first overtone of OH stretching of P-OH and PH groups, and the second overtone of 2×C=O stretching of the carboxylic acids (COOH) and esters (COOR) groups can also be found in this spectral interval. As for 7360 cm^−1^, it is the combination of 2×CH stretching and CH bending of methyl (-CH_3_).

Therefore, it is reasonable to infer that the changes in the near-infrared region absorption connected with these groups are related to the changes in proteins and their decomposition products, and in triglycerides and their decomposition products, especially fatty acids and phospholipids, during the storage process of krill.

## 4. Conclusions

In conclusion, this work sufficiently demonstrated the feasibility of integrating near-infrared spectroscopy with machine learning for rapid determination of krill storage time, thereby enabling indirect assessment of krill quality. Through the preprocessing of NIR spectra and the optimization of model hyperparameters, the LightGBM model outperformed other models with R^2^ = 0.9882, RMSE = 0.3724, MAE = 0.2018, and MAPE = 0.0431. Furthermore, model interpretation through SHAP analysis revealed that the most influential NIR spectral features primarily corresponded to protein and lipid alterations during storage, aligning with known biochemical degradation patterns in krill. Overall, the study provided an efficient and rapid method to evaluate Antarctic krill quality and offers potential for real-world use.

## Figures and Tables

**Table 1:** Modeling results of four machine learning models with different spectral preprocessing approaches.

Model	Preprocess	R^2^	RMSE	MAE	MAPE
PLSR	Raw	0.1917	3.0800	2.6571	0.8505
	MSC	0.5895	2.1949	1.7490	0.5652
	SNV	0.5869	2.2018	1.7579	0.5647
	D2	0.8209	1.4497	1.2179	0.3065
	D2+SG	0.8211	1.4489	1.2060	0.2889
	D2+MSC	0.8016	1.5260	1.2391	0.3649
	D2+SNV	0.8016	1.5258	1.2929	0.3468
SVR	Raw	0.1550	3.1492	2.5274	0.9071
	MSC	0.0530	3.3339	2.8820	0.8304
	SNV	0.0529	3.3339	2.8821	0.8304
	D2	0.8189	1.4580	1.1446	0.3752
	D2+SG	0.6521	2.0208	1.5529	0.5267
	D2+MSC	0.8239	1.4374	1.1276	0.3611
	D2+SNV	0.8240	1.4372	1.1275	0.3610
RF	Raw	0.5843	2.2089	1.6499	0.5742
	MSC	0.8096	1.4949	0.7728	0.2659
	SNV	0.8115	1.4874	0.7431	0.2567
	D2	0.9732	0.5612	0.2563	0.0547
	D2+SG	0.9862	0.4027	0.1938	0.0415
	D2+MSC	0.9419	0.8255	0.3325	0.0667
	D2+SNV	0.9438	0.8122	0.3273	0.0670
LightGBM	Raw	0.6134	2.1301	1.6006	0.5285
	MSC	0.8352	1.3907	0.7733	0.2723
	SNV	0.8549	1.3049	0.7459	0.2602
	D2	0.9823	0.4561	0.2513	0.0586
	D2+SG	0.9864	0.3999	0.2095	0.0436
	D2+MSC	0.9782	0.5063	0.3016	0.0705
	D2+SNV	0.9774	0.5154	0.3115	0.0763

## Data Availability

The original contributions presented in this study are included in the article and Supplementary Materials. Further inquiries can be directed to the corresponding authors.

## Supplementary Materials

The following supporting information can be downloaded at: https://www.mdpi.com/article/10.3390/foods14081293/s1, Figure S1: NIR spectra after treatment with different preprocessing methods ((a) MSC; (b) SNV; (c) D2; (d) D2+SG; (e) D2+MSC; (f) D2+SNV); Figure S2: Visualization of hyperparameter optimization of the machine learning models based on the Optuna algorithm. (A) Optimization history plot of PLSR. (B) Contour plot of PLSR. (C) Optimization history plot of SVR. (D) Contour plot of SVR. (E) Optimization history plot of RF. (F) Contour plot of RF. (G) Optimization history plot of LightGBM. (H) Contour plot of LightGBM; Table S1: The hyperparameter optimization routines of four models.

## Abbreviations

The following abbreviations are used in this manuscript:

NIRS	near-infrared spectroscopy
NIR	near-infrared
LightGBM	light gradient boosting machine
TVB-N	total volatile base nitrogen
NPN	non-protein nitrogen
PL	phospholipids
FFA	free fatty acid
EPA	eicosapentaenoic acid
DHA	docosahexaenoic acid
ML	machine learning
PLSR	partial least squares regression
SVR	support vector regression
RF	random forest
XAI	explainable artificial intelligence
TCA	trichloroacetic acid
SD	standard deviation
MSC	multivariate scattering correction
SNV	standard normal variable
SG	Savitzky–Golay smoothing
D2	second-order derivative
PCR	principal component regression
SVM	support vector machines
R^2^	coefficient of determination
RMSE	root mean square error
MAE	mean absolute error
MAPE	mean absolute percentage error
