# Supplementary material for: Near-Infrared Spectroscopy Combined with Explainable Machine Learning for Storage Time Prediction of Frozen Antarctic Krill
Source: Foods. 2025 Apr 8;14(8):1293. doi: 10.3390/foods14081293 (PMC12026957; doi:10.3390/foods14081293)
Supplement: Supplementary file 1 [file foods-14-01293-s001.zip › foods-3550289-supplementary.pdf]

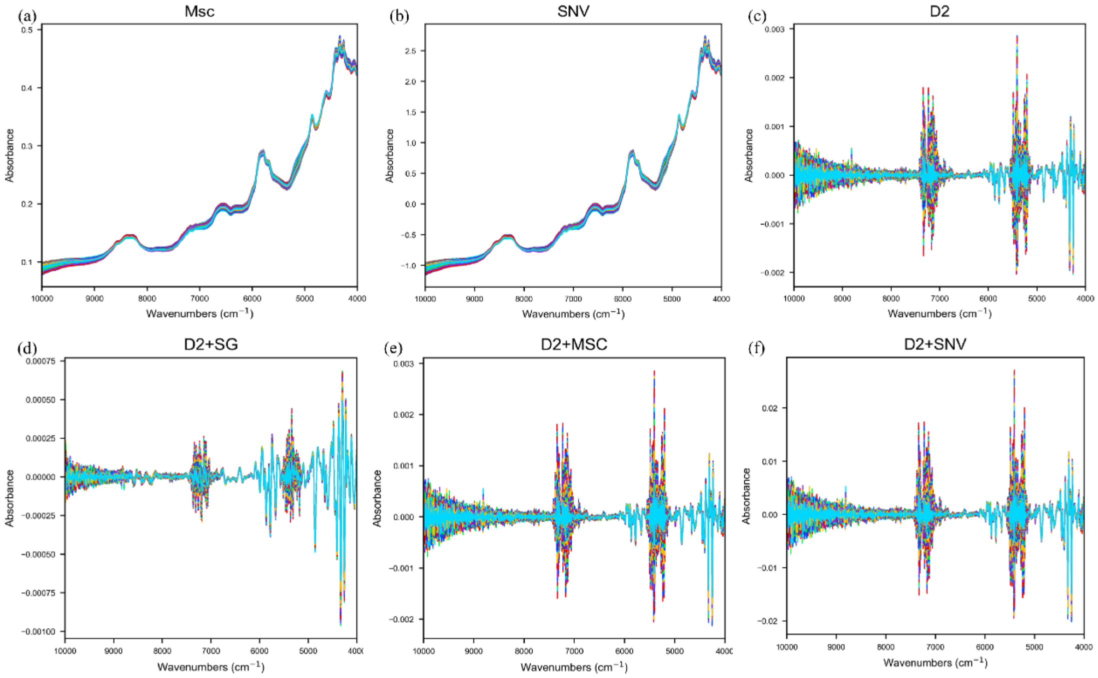

Figure S1. NIR spectra after treatment with different preprocessing methods (**a**. MSC; **b**. SNV; **c**. D2; **d**. D2+SG; **e**. D2+MSC; **f**. D2+SNV)

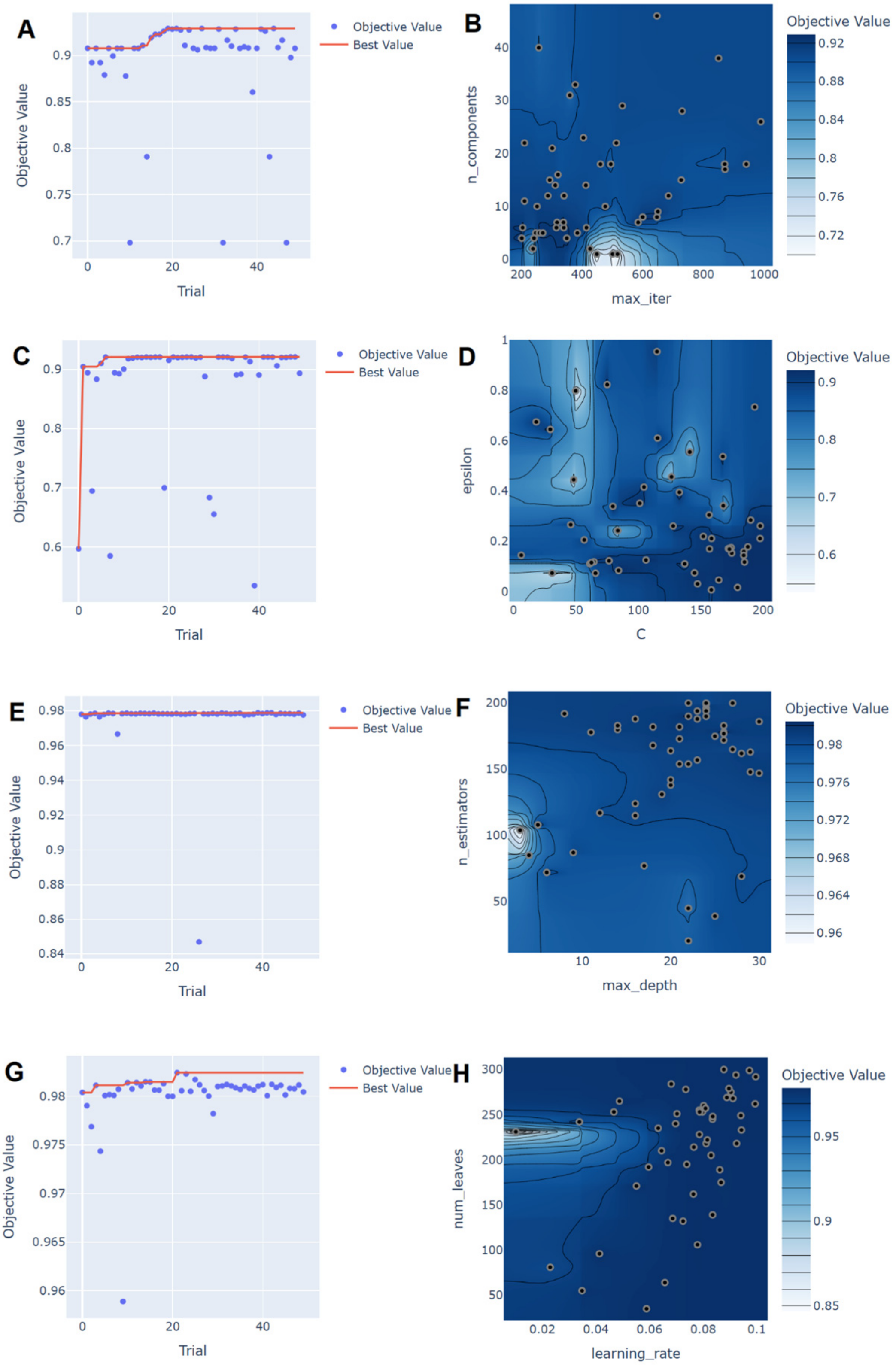

Figure S2. Visualization of hyperparameter optimization of the machine learning models based on the Optuna algorithm. (**A**: Optimization history plot of PLSR; **B**: Contour plot of PLSR; **C**: Optimization history plot of SVR; **D**: Contour plot of SVR; **E**: Optimization history plot of RF; **F**: Contour plot of RF; **G**: Optimization history plot of LightGBM; **H**: Contour plot of LightGBM）.

Table S1. The hyperparameter optimization routines of four models

Model	Hyperparameters	Default values	Tuning scope	Optimal value
	n_componnets	2	[1,10]	6
PLSR	max_iter	500	[100, 1000]	361
	Scale	True	[True, False]	True
	kernel	rbf	[rbf, linear, ploy]	rbf
SVR	epsilon	0.1	[0.1, 1]	0.1764
	C	1	[1, 200]	75.5277
	n_estimators	100	[10, 200]	171
RF	max_depth	None	[1,30]	10
	max_features	auto	[0.1,1]	0.5185
	n_estimators	100	[1, 300]	100
LightGBM	learning_rate	0.1	[0.01, 0.1]	0.0787
	num_leaves	31	[10,100]	82
